# Long-Term Use of Non-tunneled Centrally Inserted Central Venous Catheters for Home Parenteral Nutrition in Bedridden Patients: A Retrospective Cohort Study

**DOI:** 10.7759/cureus.115881

**Published:** 2026-09-07

**Authors:** Mariko Oniwa, Osamu Isono, Yuki Kataoka

**Affiliations:** 1 Department of Internal Medicine, Kyoto Min-iren Asukai Hospital, Kyoto, JPN; 2 Department of Healthcare Epidemiology, Center for Postgraduate Clinical Training and Career Development, Nagoya University Hospital, Nagoya, JPN; 3 Department of Healthcare Epidemiology, Center for Medical Education, Graduate School of Medicine, Nagoya University, Nagoya, JPN; 4 Department of Systematic Reviewers, Scientific Research Works Peer Support Group, Osaka, JPN; 5 Department of Healthcare Epidemiology, Kyoto University Graduate School of Medicine/School of Public Health, Kyoto, JPN; 6 Department of International and Community Oral Health, Tohoku University Graduate School of Dentistry, Sendai, JPN

**Keywords:** bedridden persons, catheter-related thrombosis, central line-associated bloodstream infection, home parenteral nutrition, non-tunneled centrally inserted cvcs

## Abstract

Background

Tunneled or implanted central venous catheters (CVCs) are recommended for long-term home parenteral nutrition (HPN), but some bedridden patients refuse to undergo surgical insertion. Although recent studies have demonstrated that peripherally inserted central catheters (PICCs) can be safely maintained for long-term HPN, they may also be impractical owing to patient or provider limitations. Non-tunneled, non-implanted centrally inserted CVCs (CICCs) offer bedside insertion; however, evidence on their long-term safety in home care remains limited.

Methods

We conducted a retrospective cohort study at a single home-visit center in Kyoto, Japan, enrolling patients who received HPN via CICC or PICC between January 2013 and June 2024. Patients with cancer expected to have short-term survival were excluded. The primary outcomes were central line-associated bloodstream infection (CLABSI) and catheter-related thrombosis (CRT). We calculated incidence rates per 1,000 catheter-days, estimated hazard ratios using Cox proportional hazards analysis, and compared CLABSI-free survival using Kaplan-Meier curves.

Results

A total of 16 patients (CICC: n=12; PICC: n=4) contributed 2,694 catheter-days of observation. Six CLABSI events occurred (CICC: 4; PICC: 2). The CLABSI incidence rate was 1.79 per 1,000 catheter-days in the CICC group and 4.36 in the PICC group (hazard ratio, 8.65; 95% CI, 0.76-98.8; P=0.082). No CRT events occurred. At six months, CLABSI-free survival was 91.7% for CICCs and 50.0% for PICCs.

Conclusions

For bedridden patients for whom tunneled or implanted CVCs or PICCs are not feasible, CICCs may represent a potential option for long-term HPN. However, the small sample size, limited number of outcome events, and lack of multivariable adjustment warrant cautious interpretation and further study.

## Introduction

Home parenteral nutrition (HPN) using central venous catheters (CVCs) is an established therapy for patients requiring long-term parenteral nutritional support outside the hospital. It improves survival outcomes by maintaining nutritional status, enhances quality of life (QOL) by reducing hospital admissions, and lowers healthcare costs. In the United States, more than 25,000 patients (79 per million) were estimated to receive HPN in 2013, and according to claims data obtained by the National Home Infusion Association (NHIA), the number of patients receiving HPN annually was approximately 32,000 from 2022 to 2024 [1,2].

The CVCs used for parenteral nutrition include implanted ports, tunneled catheters, peripherally inserted central catheters (PICCs), and non-tunneled, non-implanted centrally inserted CVCs (CICCs). Implanted or tunneled CVCs are recommended for long-term use because of their lower infection risks, but they require surgical insertion. Both PICCs and CICCs can be inserted at the bedside. While PICC lines were previously recommended for use over a period of several months, recent studies have demonstrated that PICCs can be safely maintained for long-term HPN, with the potential to remain in place for several years without major complications [3]. The CICC lines are used for short-term or acute-phase treatment. This is because they lack a subcutaneous tunnel or cuff, resulting in weaker infection-prevention capabilities, and inadequate fixation increases the risk of puncture-site damage and infection.

Implanted or tunneled catheters and PICCs are recommended for HPN. However, no reports have evaluated the safety or feasibility of long-term CICC placement. Some bedridden patients refuse surgery for implanted or tunneled catheters. The PICC placement at home may also be difficult due to patient-related issues (e.g., severe contractures) or provider-related issues (e.g., lack of home ultrasound access or skilled personnel). The CICCs offer potential advantages in home care settings: they can be inserted at the bedside without surgical intervention, the insertion technique is widely familiar to physicians in various clinical settings, and catheter exchange can be performed relatively easily when needed. With diligent infection control, CICCs might serve as a viable option for long-term HPN in immobile patients. The primary objective of this study was to evaluate the safety of long-term CICC use in HPN by assessing the incidence and time to catheter-related complications, particularly central line-associated bloodstream infection (CLABSI). The secondary objective was to evaluate feasibility by investigating catheter duration and reasons for catheter removal and by comparing these outcomes with those of PICCs. To address these objectives, we retrospectively reviewed our institutional experience with CICC and PICC use for HPN in patients receiving home medical care.

## Materials and methods

Study design and participants

This study was approved by the Ethics Committee of the Kyoto Min-iren Asukai Hospital (approval no. 202411-02). Because of the retrospective nature of the study and the use of anonymized data, the requirement for individual informed consent was waived, and an opt-out approach was adopted. We employed a retrospective cohort design based on a detailed medical chart review to evaluate the incidence of and time to catheter-related complications during long-term HPN in routine clinical practice. A randomized controlled trial was impractical because long-term HPN is used in a limited number of patients, making it difficult to accrue an adequate sample size within a feasible period. A claims-based study was also unsuitable because claims data do not capture clinical details such as catheter lumen type. This design enabled a longitudinal evaluation of catheter-related outcomes using detailed clinical data collected in home-care settings. We report this study following the Strengthening the Reporting of Observational Studies in Epidemiology (STROBE) statement [4].

The study subjects were patients who were managed at the Home Visit Center of Kyoto Min-iren Asukai Hospital (Kyoto, JPN) between 1 January 2013 and 30 June 2024, who had a CICC or PICC with regulatory management by visiting nurses, were treated with intravenous hyperalimentation at home, and received a Japanese home central venous nutrition instruction and management fee (billing code 114004210). Consecutive sampling was used, and all eligible patients during the study period were included. Patients with advanced cancer were excluded because their limited life expectancy could substantially affect catheter duration and complication rates. For CICCs, catheter insertion was performed using either static ultrasound location or real-time ultrasound guidance; for PICCs, insertion was performed using real-time ultrasound guidance, and catheter tip position was confirmed by fluoroscopy. When catheter replacement was necessary due to catheter malposition, dislodgement, or suspected catheter-related complications, it was performed either as an outpatient procedure or during hospitalization.

Variables and definitions

We extracted data from medical records, including catheter type, patient age, sex, BMI, activities of daily living (ADL), catheter lumen type, nutritional route, and catheter duration. The ADL was categorized into three levels: (1) able to perform daily activities without assistance, (2) requiring some assistance for indoor activities, and (3) bedridden and dependent for toileting, eating, and dressing. Age, sex, BMI, ADL status, catheter lumen type, and route of nutrition were considered as potential confounding variables that might influence the risk of catheter-related complications. However, because the number of outcome events was limited, no multivariate adjustment was performed.

Outcome measures

We investigated CLABSI and catheter-related thrombosis (CRT) as complications associated with central venous catheters, analyzing the time to onset. We defined CLABSI as a bloodstream infection occurring in patients with a central venous catheter in situ for more than 48 hours, after excluding other potential sources of infection (e.g., urinary tract infection, pneumonia) [5]. We defined CRT as any thrombotic event associated with an indwelling central venous catheter [6]. Follow-up and data accrual were completed on April 10, 2025, representing the maximum follow-up period after the end of enrollment (June 30, 2024). The unit of analysis was individual catheter insertion episodes rather than individual patients. Each episode was defined as the period from catheter insertion to catheter removal, death, or the end of the observation period, whichever came first. Although some patients contributed more than one catheter insertion episode, models accounting for within-patient correlation were not applied due to the small number of patients and outcome events.

Bias control

To minimize selection bias and information bias, infection was determined by one physician reviewing the chart for blood cultures and clinical findings and consulting with a second physician if the decision was difficult. To address selection bias, we included all eligible consecutive patients who received treatment during the study period.

Statistical analysis

We did not calculate the formal sample size because of the exploratory nature of this retrospective study and the limited number of eligible patients, and we analyzed all available cases during the study period. We calculated complication incidence rates for each group and estimated hazard ratios using univariable Cox proportional hazards regression [7]. Kaplan-Meier survival analysis [8] was performed to compare the duration of complication-free survival. All statistical analyses were conducted using Python software version 3.11.8 (Python Software Foundation, Wilmington, Delaware, USA). All variables were complete; therefore, we conducted a complete case analysis. Multivariable models were planned but not fitted because the total number of outcome events (<10) precluded reliable adjustment.

## Results

Participants

From January 1, 2013, to June 30, 2024, there were 18 patients who had received home visits from our hospital and had either a CICC or a PICC. Of these, we excluded two patients who were predicted to die in the short term due to cancer. Twelve patients had a CICC, and four had a PICC (Figure 1). Throughout the study, the catheter was locked when necessary with 100 U/ml heparin and not with antibiotics or ethanol.

**Figure 1:**
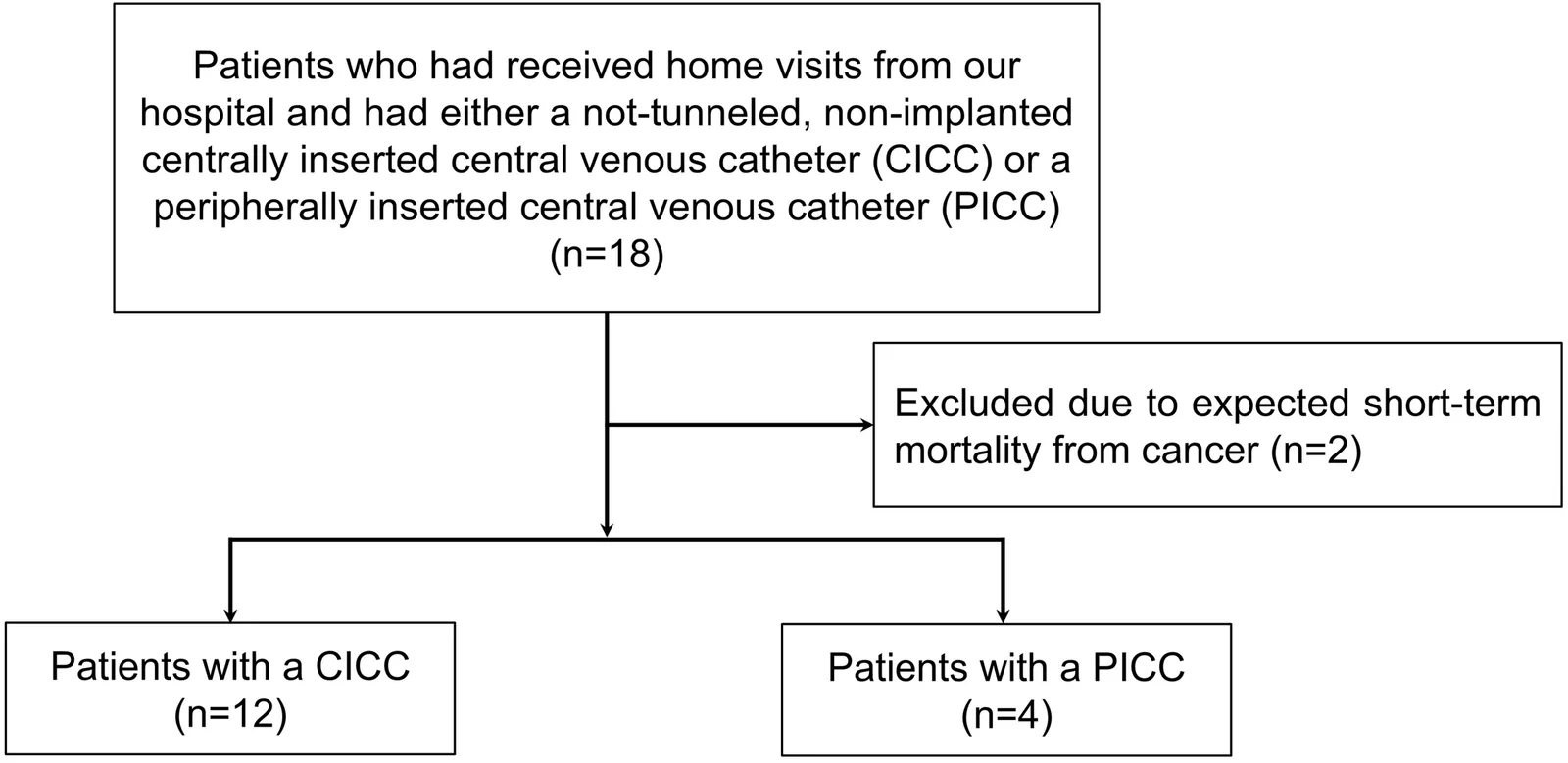
Flow diagram of participant selection CICC: Non-implanted centrally inserted central venous catheters; PICC: Peripherally inserted central catheters

Descriptive data

There were 16 eligible patients with a total follow-up of 2,694 catheter days and a median follow-up of 144 days (IQR 47-270) (Table 1). Baseline characteristics are summarized in Table 2. Patients in the CICC group were older than those in the PICC group and were predominantly female. They also had a higher mean BMI and a longer mean catheter dwell time. In contrast, patients in the PICC group were younger, had a lower mean BMI, and had a shorter mean catheter indwell time. Oral intake was maintained in half of the patients in both groups. The ADL also differed between groups, with most patients in the CICC group being bedridden, whereas patients in the PICC group generally had functional independence with some assistance.

**Table 1:** Reasons for removal in patients with CICC and PICC Values are presented as individual catheter episodes. CICC: Non-tunneled centrally inserted central venous catheter; PICC: Peripherally inserted central catheter; CLABSI: Central line-associated bloodstream infection; MRCNS: Methicillin-resistant coagulase-negative staphylococci; MRSA: Methicillin-resistant *Staphylococcus aureus*

Patient	Catheter type	Duration (days)	Reason for removal	Causative pathogen
1	CICC	47	Dislodgement	—
	CICC	296	CLABSI	MRCNS
	CICC	354	CLABSI	Candida parapsilosis
	CICC	261	Death	—
2	CICC	47	Dislodgement	—
3	CICC	382	Catheter malposition	—
	CICC	330	CLABSI	Not identified
	CICC	113	Death	—
4	CICC	141	Conversion to implanted port	—
5	CICC	17	CLABSI	MRSA
	CICC	148	Conversion to gastrostomy	—
6	CICC	99	Death	—
7	PICC	258	CLABSI	Trichosporon asahii
8	PICC	15	Death	—
9	PICC	14	Dislodgement	—
10	PICC	172	CLABSI	Candida parapsilosis

**Table 2:** Baseline characteristics of patients by catheter type at initial catheter placement Values are presented as mean ± standard deviation or number (%). *Lumen type data were missing for three patients in the CICC group; percentages for lumen type were calculated among patients with available data. No missing data were observed for age, sex, BMI, nutrition route, comorbidities, or catheter duration. CICC: Non-tunneled centrally inserted central venous catheter; PICC: Peripherally inserted central catheter; ADL: Activities of daily iving

Variable		CICC group (n = 12)	PICC group (n = 4)
Age (years)		90.7 ± 8.5	70.5 ± 6.4
Sex (male/female)		3 (25.0) / 9 (75.0)	2 (50.0) / 2 (50.0)
BMI, kg/m²		18.1 ± 2.9	15.2 ± 1.6
ADL	Able to perform daily activities without assistance	0 (0)	0 (0)
	Requires some assistance for indoor activities	2 (16.7)	3 (75.0)
	Bedridden and dependent for toileting, eating, and dressing	10 (83.3)	1 (25.0)
Lumen type, external diameter *	Single lumen, 1.5 mm	8 (88.9)	-
	Double lumen, 1.7 mm	1 (11.1)	-
	Single lumen, 1.0 mm	-	3 (75.0)
	Double lumen, 1.3 mm	-	1 (25.0)
Catheter site	The internal jugular vein	11 (91.7)	-
	The femoral vein	1 (8.3)	-
	The basilic vein	-	4 (100.0)
Nutrition route	Oral	6 (50.0)	2 (50.0)
	Non-oral	6 (50.0)	2 (50.0)
Comorbidities	Neutropenia	0 (0)	0 (0)
	Malignancy	0 (0)	0 (0)
	History of sepsis	1 (8.3)	0 (0)
	Chronic kidney disease	4 (33.3)	1 (25.0)
	Previous venous thromboembolism	0 (0)	0 (0)
	Coagulation disorders	0 (0)	0 (0)
	Anticoagulant or antiplatelet medication use	5 (41.7)	0 (0)

Clinical outcomes

Patients in the CICC group were older than those in the PICC group, as mentioned before; catheter duration and complication rates are shown in Table 3. Among 16 eligible patients, six cases (37.5%) developed CLABSI, whereas no cases of catheter-related thrombosis were identified. The CLABSI occurred more frequently in the PICC group than in the CICC group, with incidence rates of 4.36 and 1.79 per 1,000 catheter-days, respectively. The univariable Cox regression yielded a hazard ratio of 8.65 for CLABSI for PICCs compared with CICCs, although the confidence interval was wide and the difference did not reach statistical significance. Multivariable analysis was not performed because the limited number of outcome events precluded reliable adjustment.

**Table 3:** Catheter duration, CLABSI incidence, and hazard ratios by catheter type Catheter duration is presented as mean ± standard deviation. Incidence rates are expressed as events per 1,000 catheter-days. Total catheter duration was 2,235 catheter-days in the CICC group and 459 catheter-days in the PICC group. Hazard ratios were estimated using univariable Cox proportional hazards regression. All p-values were two-sided, and P <0.05 was considered statistically significant. CLABSI: Central line-associated bloodstream infection; CICC: Non-tunneled centrally inserted central venous catheter; PICC: Peripherally inserted central catheter

Catheter type	Catheter duration (days)	CLABSI events (n)	Incidence rate (per 1,000 catheter-days)	Hazard ratio (95% CI)	p-value
CICC	185.8 ± 116.4	4	1.79	Reference	—
PICC	114.8 ± 117.6	2	4.36	8.65 (0.76–98.8)	0.082

Kaplan-Meier analysis further revealed differences in the timing of CLABSI between catheter types (Figure 2). In patients with PICC lines, infections occurred earlier in the follow-up period, resulting in a steeper decline in the CLABSI-free survival curve. In contrast, the CICC group maintained a high probability of remaining CLABSI-free throughout most of the observation period. The estimated CLABSI-free survival rate at six months was 91.7% in the CICC group and 50.0% in the PICC group; however, these estimates should be interpreted cautiously given the small sample size.

**Figure 2:**
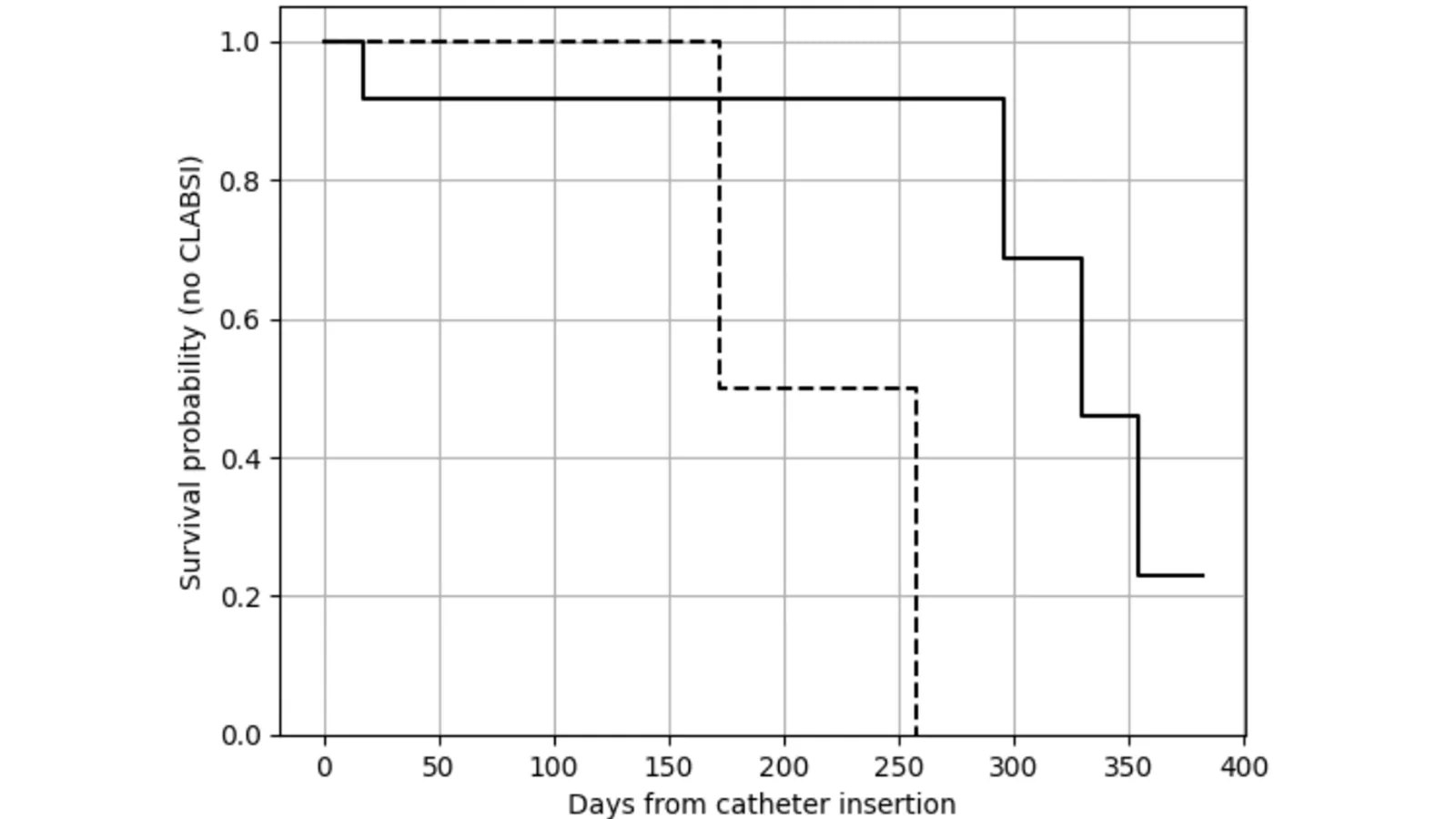
Kaplan-Meier CLABSI-free survival by device type The solid line indicates CICCs and the dashed line indicates PICCs. The CLABSI was treated as the event, and non-CLABSI catheter removal as censoring. CLABSI: Central line-associated bloodstream infection; CICC: Non-tunneled centrally inserted central venous catheter; PICC: Peripherally inserted central catheter

## Discussion

In this retrospective home-care cohort study of patients with severely limited mobility, the CLABSI incidence rate was 1.79 per 1000 catheter-days in the CICC group and 4.36 per 1000 catheter-days in the PICC group. The median time to CLABSI was 312.5 days (range: 16-354 days) in the CICC group and 205 days (range: 168-242 days) in the PICC group. The CRT incidence rate was 0 in both groups.

The CICC may warrant further evaluation for long-term HPN in selected patients with limited ADL under appropriate catheter management. According to the European Society for Clinical Nutrition and Metabolism (ESPEN) guidelines, tunneled or fully implanted CVCs are recommended for long-term HPN, while PICCs are only suitable for short-term use (less than three months) [9]. However, in fact, several studies have demonstrated that PICCs can be safely maintained for long-term HPN, and the proportion of patients receiving PICC lines is increasing. In Canada, for 2011-2014 compared with 2005-2008, the use of tunneled catheters has decreased from 64.3% to 38%; in contrast, the proportion of PICCs has increased from 21.6% to 52.9% for HPN [10]. In the United States as well, from 2011 to 2014, PICC lines were the primary method of HPN administration, accounting for 47% of cases, followed by tunneled catheters at 43% and subcutaneous infusion ports at 10% [11].

The CICC is generally not recommended for long-term use due to the risk of infection and thrombosis. Regarding infection risk, the CLABSI incidence rate for adult intestinal-failure patients without malignancy using home central venous access devices with heparin locks (subcutaneous tunneled central venous catheters or subcutaneous port systems) has been reported as 2.01 per 1,000 catheter days [12]. Furthermore, a recent report indicated that the median time to catheter-related bloodstream infection (CRBSI) occurrence in non-cancer adult patients with intestinal failure using a heparin lock-equipped tunneled CVC for HPN therapy was 154 days (range: 87-438 days) [13].

Regarding thrombosis risk, past cohort studies reported a CRT incidence rate of 0.12 per 1000 catheter days in non-cancer adult patients with HPN using tunneled or implanted CVCs, and this incidence was unrelated to the use of saline or heparin lock solutions [14]. The CRT incidence rate for PICC or tunneled catheters was reported as 0.08 per 1,000 catheter days in adult patients with inflammatory bowel disease (IBD) and 0.12 per 1,000 catheter days in patients with intestinal dysfunction (IF), though the locking method was not specified [15,16]. While previous studies did not document ADL status, most patients in this study were bedridden. Immobility is a recognized patient-related risk factor for catheter-related thrombosis [17]. Although immobility itself has not been clearly established as an independent risk factor for CRBSI, it may indirectly increase infection risk through impaired self-care and hygiene. Regarding CLABSI and CRT rates, the incidence rates observed with long-term CICC use in this study were within the ranges reported in previous studies of tunneled, implanted, and PICC devices used for HPN. The higher CLABSI incidence in the PICC group in this study may be due to the small number of PICC cases and the fact that home care providers were not yet accustomed to managing PICCs. These findings support further investigation of long-term CICC use in patients unable to use other CVCs.

The CICCs are generally used in acute or emergency settings, but they have the potential to expand treatment options for home-based patients requiring central venous nutrition. Tunneled and implanted central venous catheters, recommended for long-term HPN, require surgical placement under sedation by general surgeons, vascular surgeons, or interventional radiologists [18]. The PICC lines have also been suggested to allow for long-term HPN use [19,20]. In Japan, PICC use has recently expanded, and certified nurses have been authorized to perform insertions [21]. But this is limited to hospitals with training facilities. In general hospitals, the number of physicians capable of inserting PICCs remains limited, and home nursing and home care services are still unfamiliar with PICC use. To promote the use of PICC lines in home care settings, it is desirable to expand training to general clinics and nursing stations that support home care. Meanwhile, CICCs have long been established as a device for acute care settings. These findings raise the possibility that, if supported by further research, CICC use could provide an additional approach to HPN for patients who do not wish to undergo the invasive procedure for implantable or tunneled CVCs, as well as for patients in areas where PICC use is difficult.

This study has several limitations. First, the number of cases was small, which limits the statistical power and generalizability of the findings. Additionally, there was a marked imbalance in the number of patients between the CICC and PICC groups, and substantial differences were observed in baseline characteristics such as age, BMI, and ADL. These differences may have introduced confounding factors, making it difficult to attribute the observed differences in catheter-related outcomes solely to catheter type. Multivariate adjustment was not performed due to the low number of outcome events. Moreover, the wide confidence interval for the hazard ratio (HR: 8.65, 95% CI: 0.76-98.8) for CLABSI indicates considerable statistical uncertainty. Therefore, the findings should be interpreted as exploratory and hypothesis-generating rather than as definitive evidence of the superiority of CICC over PICC. Second, this is a single-center, retrospective study, and the results may not be generalizable to other home care settings or patient populations. Third, a standardized catheter management protocol was not implemented for all cases, and variations in insertion techniques and catheter care may have influenced the incidence rates of CLABSI and CRT. Future multicenter prospective studies with larger sample sizes, more balanced patient cohorts, and standardized processes, such as checklists for infection prevention and aseptic technique, are needed to further evaluate the safety and feasibility of long-term CICC use in the home setting.

## Conclusions

In this retrospective cohort study of bedridden patients receiving HPN, CLABSI and CRT rates for long-term use of CICCs were within the range previously reported for tunneled or implanted CVCs and PICCs used for HPN. Although CICCs are generally intended for short-term use, these findings provide preliminary real-world data on their use for long-term HPN in home-care settings. Given the small sample size, imbalance between the CICC and PICC groups, differences in baseline characteristics, and lack of multivariable adjustment, further multicenter prospective studies with larger and more balanced patient populations are needed to determine the safety and feasibility of long-term CICC use in selected patients receiving HPN. If supported by larger studies, long-term CICC use could potentially provide an additional approach to HPN for patients with limited treatment options and may increase flexibility in community-based care.
